# DESIGNING STATE-BASED LTSS PROGRAMS IN CONTEXT OF UNIVERSAL FAMILY CARE AND EMANCIPATORY GERONTOLOGY

**DOI:** 10.1093/geroni/igz038.2121

**Published:** 2019-11-08

**Authors:** Benjamin Veghte, Carroll Estes, Stacy Torres

**Affiliations:** 1 National Academy of Social Insurance, Washington, District of Columbia, United States; 2 University of California, San Francisco, San Francisco, California, United States

## Abstract

This symposium will present findings from a National Academy of Social Insurance study panel on Designing State-Based Social Insurance Programs for Long-Term Services and Supports, Paid Leave, and Affordable Child Care. The risk of needing to provide or receive care is universal. Policymakers in several states are now weighing the enactment of new social insurance programs to address the risk of needing long-term services and supports. The study panel has mapped out the key design choices such states would need to consider with regard to program structure, financing, integration with Medicaid, and implementation, and the implications of these choices for elders, people with disabilities, families, providers, and states. The symposium will also present the study panel’s findings with regard to how long-term care benefits could be provided in the context of an integrated care program addressing three often interrelated caregiving risks: long-term services and supports, paid family and medical leave, and early child care and education: Universal Family Care. Finally, implications for emancipatory gerontology, including the impact of the current gaps in our care infrastructure on family caregivers and the care workforce, will be considered.

